# Expression of L-Amino Acid Oxidase (Ml-LAAO) from the Venom of the *Micrurus lemniscatus* Snake in a Mammalian Cell System

**DOI:** 10.3390/toxins17100491

**Published:** 2025-10-02

**Authors:** Ari Junio de Oliveira Costa, Alessandra Matavel, Patricia Cota Campos, Jaqueline Leal dos Santos, Ana Caroline Zampiroli Ataide, Sophie Yvette Leclercq, Valéria Gonçalves de Alvarenga, Sergio Caldas, William Castro-Borges, Márcia Helena Borges

**Affiliations:** 1 Laboratório de Proteômica e aracnídeos, Diretoria de Pesquisa e Desenvolvimento, Fundação Ezequiel Dias-Rua Conde Pereira Carneiro 80, Gameleira, Belo Horizonte 30510-010, Minas Gerais, Brazil; aryjunio1998@gmail.com (A.J.d.O.C.); jaqueline.leal@funed.mg.gov.br (J.L.d.S.); 2 Serviço de Toxinologia Molecular, Diretoria de Pesquisa e Desenvolvimento, Fundação Ezequiel Dias-Rua Conde Pereira Carneiro 80, Gameleira, Belo Horizonte 30510-010, Minas Gerais, Brazil; matavela@yahoo.com.br; 3 Serviço de Proteômica e Aracnídeos, Diretoria de Pesquisa e Desenvolvimento, Fundação Ezequiel Dias-Rua Conde Pereira Carneiro 80, Gameleira, Belo Horizonte 30510-010, Minas Gerais, Brazil; pcota2013@gmail.com; 4 Laboratório de Pesquisa em Doenças Infecciosas, Diretoria de Pesquisa e Desenvolvimento, Fundação Ezequiel Dias-Rua Conde Pereira Carneiro 80, Gameleira, Belo Horizonte 30510-010, Minas Gerais, Brazil; ana.ataide@funed.mg.gov.br (A.C.Z.A.); sergio.caldas@funed.mg.gov.br (S.C.); 5 Laboratório Desenvolvimento de Produtos Biológicos, Diretoria Industrial, Fundação Ezequiel Dias-Rua Conde Pereira Carneiro 80, Gameleira, Belo Horizonte 30510-010, Minas Gerais, Brazil; sophie.leclercq@funed.mg.gov.br; 6 Laboratório de Bioquímica de Venenos Animais, Diretoria de Pesquisa e Desenvolvimento, Fundação Ezequiel Dias-Rua Conde Pereira Carneiro 80, Gameleira, Belo Horizonte 30510-010, Minas Gerais, Brazil; valeria.alvarenga@funed.mg.gov.br; 7 Laboratório de Enzimologia e Proteômica-Universidade Federal de Ouro Preto-UFOP, Ouro Preto 35400-000, Minas Gerais, Brazil; wborges@ufop.edu.br

**Keywords:** L-amino acid oxidase, *Micrurus lemniscatus*, recombinant protein, HEK293T cell

## Abstract

Animal venoms are rich in bioactive molecules with promising biotechnological potential. They comprise both protein and non-protein toxins. Among the protein toxins are enzymes, such as phospholipases A_2_, proteases and L-amino acid oxidases (LAAOs). LAAOs exhibit antimicrobial, antiparasitic, antiviral, and anticancer effects, making them potential candidates for biotechnological applications. These activities are linked to their ability to catalyze oxidative reactions that convert L-amino acids into α-keto acids, releasing ammonia and hydrogen peroxide, which contribute to the immune response, pathogen elimination, and oxidative stress. However, in snakes of the *Micrurus* genus, LAAOs generally represent a small portion of the venom (up to ~7%), which limits their isolation and study. To overcome this, the present study aimed to produce Ml-LAAO, the enzyme from *Micrurus lemniscatus*, through heterologous expression in mammalian cells. The gene sequence was inferred from its primary structure and synthesized into the pSecTag2B vector for expression in HEK293T cells. After purification using a His Trap-HP column, the presence of recombinant Ml-LAAO (Ml-LAAOrec) was confirmed by Western blot and mass spectrometry, validating its identity. These results support successful recombinant expression of Ml-LAAO and highlight its potential for scalable production and future biotechnological applications.

## 1. Introduction

Animal venoms play a pivotal role in the ecological and evolutionary adaptations of venomous species, being used for critical functions in predation, defense, and competition deterrence [1]. Among venomous animals, snakes, spiders, and scorpions are the most extensively studied due to their clinical and biotechnology importance [1]. Within the clade Serpentes, approximately 600 venomous snake species have been identified globally, distributed across four primary families: *Hydrophiidae, Viperidae, Crotalidae*, and *Elapidae* [2,3].

Envenomations caused by species from the families *Viperidae* and *Crotalidae* are often associated with severe pathophysiological effects, including intravascular coagulation, local and systemic hemorrhage, marked edema, and tissue necrosis [4]. In contrast, the venoms of *Hydrophiidae* and *Elapidae* are primarily neurotoxic, exerting their effects through the inhibition of neuromuscular transmission, often leading to paralysis [4]. This diversity in venom composition and function underscores the evolutionary specialization of these families and highlights the importance of studying their venoms for ecological understanding, potential therapeutic applications, and improved knowledge of envenomation mechanisms to enhance treatment strategies.

The *Elapidae* family, comprising approximately 416 species, exhibits a broad geographical distribution, spanning the Americas, Asia, Africa, and Australia [5]. Within this family, the genus *Micrurus*, commonly known as coral snakes, comprises venomous elapid species distributed throughout the Americas. Species of the genus *Micrurus* are generally characterized by aposematic coloration, often consisting of brightly colored rings arranged in triads or dyads, and a proportionally short tail. However, coloration patterns and tail length may vary among species. These traits have been widely described in taxonomic and herpetological literature [6,7].

Among the notable species is *Micrurus lemniscatus*, which is widely distributed across Brazil and is recognized for its potent neurotoxic venom [8,9]. The venom of *Micrurus lemniscatus* is highly complex, consisting of a diverse array of proteins, enzymes, and chemical mediators, such as histamine and serotonin [10]. The main protein classes identified include three-finger toxins (3FTxs), phospholipases A_2_ (PLA_2_s), L-amino acid oxidases (LAAOs), Kunitz-type serine protease inhibitors, and metalloproteinases, which collectively contribute to the neurotoxic and, to a lesser extent, cytotoxic effects of the venom [11]. Its neurotoxic effects, primarily mediated through the disruption of neuromuscular transmission, are well-documented [12]. Among the enzymatic components of this venom, L-amino acid oxidases (LAAOs) have attracted considerable interest due to their distinctive biological properties and promising pharmacological applications.

In general, the LAAOs of snake venoms catalyze the oxidative deamination of L-amino acids into α-keto acids, with the concomitant production of ammonia and hydrogen peroxide (H_2_O_2_) [13]. Due to H_2_O_2_ production, these enzymes have shown antimicrobial and cytotoxic activities [14,15]. Furthermore, they are known to induce platelet aggregation and modulate immune system responses [16,17].

In addition, they can induce DNA damage and apoptosis in both normal and tumor cells [18,19,20,21]. For instance, LAAO from *Agkistrodon halys* caused apoptosis and necrosis in murine leukemia cells (L1210), effects only partially reversed by catalase, highlighting mechanisms beyond H_2_O_2_ generation and anticancer potential [22,23].

Additionally, LAAOs from *Bothrops* species show strong antiparasitic activity against *Trypanosoma cruzi* [24,25], reinforcing their promise as candidates for drug development against cancer and neglected tropical diseases.

Despite their potential, LAAOs typically represent a small fraction of *Micrurus* venom, limiting their biotechnological exploration [26]. In the *Elapidae* family, LAAOs typically account for approximately 5–8% of the total venom composition. However, in *Micrurus* species, their abundance is usually lower, rarely exceeding 7% [26,27]. For instance, in *Micrurus lemniscatus*, LAAOs account for approximately 2% of the venom [28].

The advancement of recombinant expression techniques has enabled the more efficient and controlled production of proteins, including LAAOs from animal venoms, thereby overcoming the challenges associated with extracting these enzymes directly from venom [29]. Consequently, the use of expression systems for producing recombinant venom proteins is essential for the biotechnological exploration of these bioactive molecules [29,30]. Thus, the present study aimed to express L-amino acid oxidase (Ml-LAAO) from the venom of *Micrurus lemniscatus* in HEK293T mammalian cells, establishing a valuable strategy for recombinant enzyme production and enabling future opportunities for its biotechnological application.

## 2. Results and Discussion

### 2.1. Optimization and In Silico Analysis of ML-LAAOnat and ML-LAAOrec

The four fragments (totaling 52 residues) identified by Soares et al. (2020) [28] (READYEEFLEIA, RNDMEGWYVNLGPMR, LSEFVQENENAWYYIK, FWEVHGIR) were compared with entries in the UniProt and DDBJ databases.

**Figure 1:**
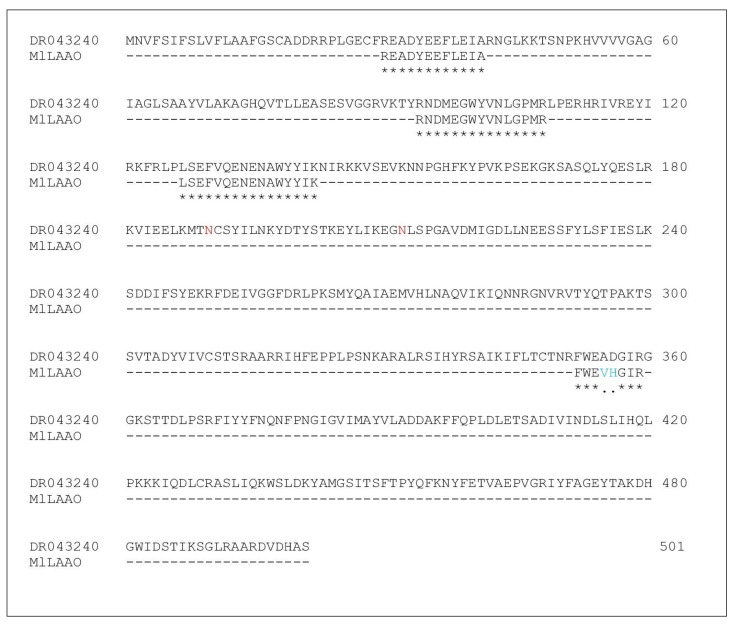
Comparative Alignment of Ml-LAAO Peptides with DDBJ Reference (DR043240), Fragments from Soares et al. (2020) [28] aligned (MAFFT v7.511) with the Ml-LAAO sequence from DDBJ. Blue-(VH) highlighted semi-conservative substitutions relative to the reference sequence (AD). Red-highlighted residues (N) predicted N-glycosylation sites (NetNGlyc). Asterisks (*) marks identical residues; single dots (.) show semi-conservative substitutions.

The sequence with the highest identity was selected from Aird et al., 2017 [31] (BioSample—SAMD00076720; bioproject: PRJDB5628, sequence: DR043240). Peptide alignments for MAFFT (v7.511) with the reference sequence (Figure 1) revealed an overall 96% similarity in the matched regions, and based on this high homology, the sequence was selected and subsequently optimized for expression in HEK293T cells.

In silico analyses of the native protein (Ml-LAAOnat) and recombinant (Ml-LAAOrec) were performed using ProtParam (https://web.expasy.org/protparam/ accessed on 26 March 2022).

(Table 1). Ml-LAAOrec, comprising 543 amino acids, includes an additional 42 residues compared to the native protein (501 amino acids). These additional residues are attributed to the inclusion of XhoI and HindIII restriction sites, as well as a poli-His tail affinity tag.

Following optimization, the recombinant protein exhibited an increase in molecular mass of approximately 5 kDa compared to its native form. Additionally, a slight shift in the isoelectric point was observed, from 8.97 to 9.02. Other physicochemical properties, such as the estimated half-life in mammalian cells, instability index, and aliphatic index (a parameter that reflects solubility in H_2_O), remained largely unchanged. Homology modeling of Ml-LAAOrec was performed using the Swiss-Model platform, based on its native Ml-LAAO. The native structure itself was previously modeled using the LAAO enzyme from the venom of the snake *Calloselasma rhodostoma* as a template [32], which showed 75.98% sequence similarity to Ml-LAAOrec. Additionally, structural prediction using Phyre2 confirmed 100% sequence identity and 96% coverage, supporting a high level of confidence in the similarity between the predicted structure and the native conformation of Ml-LAAOnat.

**Figure 2:**
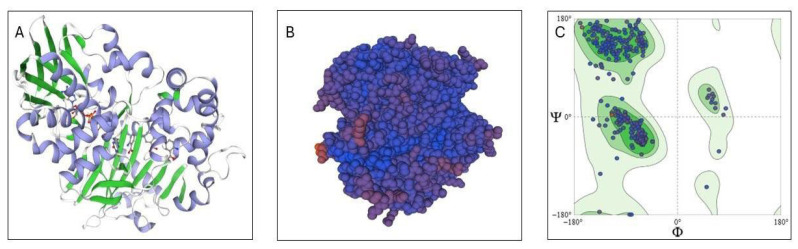
Structural and sequence-based features of Ml-LAAOrec predicted by Swiss-Model and complementary in silico tools. (**A**) Ribbon diagram of the predicted three-dimensional structure, highlighting β-sheets (green) and α-helices (blue). (**B**) Surface (spacefill) representation colored according to sequence conservation, with highly conserved residues in dark blue, and less conserved residues in gradients ranging from purple to red. (**C**) Ramachandran plot illustrating the distribution of backbone dihedral angles Φ (phi) and Ψ (psi). Green corresponds to energetically favorable conformations, and blue dots represent individual residues. The most populated regions correspond to α-helices (Φ ≈ −60°, Ψ ≈ −40°) and β-sheets (Φ ≈ −120°, Ψ ≈ 120°).

According to the algorithm, the secondary structure of Ml-LAAOrec comprises approximately 28% α-helices, 21% β-sheets, and 4% disordered regions (Figure 2A). Surface mapping revealed solvent-accessible areas and potential interaction sites (Figure 2B). The stereochemical quality of the model was validated using a Ramachandran plot (Figure 2C), which indicated a distribution of torsion angles consistent with a stable and stereochemically reliable conformation.

As expected for a secreted protein, SignalP analysis of the Ml-LAAOnat sequence deposited in the DDBJ database revealed the presence of a signal peptide. The algorithm predicted a cleavage site between residues Cys18 and Ala19 with a high probability score (98.7%) (Figure S1A), which is likely cleaved by signal peptidase I. This prediction was subsequently validated through Protter analysis (Figure S1B).

NetNGlyc predicted a 65.1% probability of N-linked glycosylation at Asn190 (NCSY). Although Soares (2020) [28] did not identify the exact glycosylation site, the experimental data supported the occurrence of N-glycosylation, as evidenced by bands around ~69 kDa before and ~44 kDa after cleavage. An additional potential glycosylation site was predicted at Asn 228 (NLSP), with a lower probability of 16.1%. All predicted sites were mapped using Protter.

### 2.2. Expression Optimization

**Figure 3:**
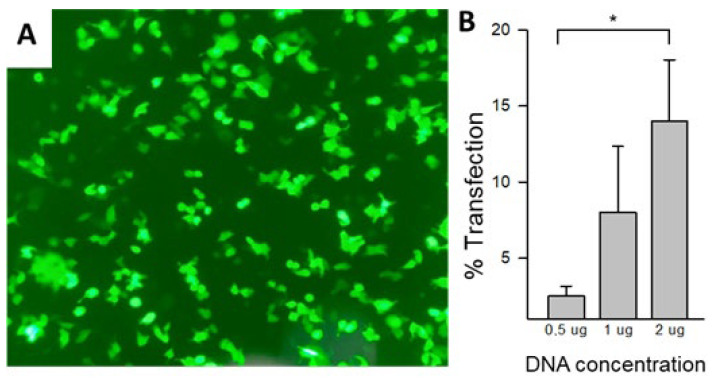
Standardization of Ml-LAAOrec expression in HEK293 cells. (**A**) Fluorescence microscopy (SDP-TOP/ICX41) of HEK293 cells co-transfected with Ml-LAAOrec DNA (1 µg) and EGFP (0.2 µg). (**B**) Percentage of transfected cells at different Ml-LAAOrec DNA concentrations (0.5, 1, and 2 µg) co-transfected with 0.2 µg EGFP, quantified using a Countess 3FL automated cell counter (Invitrogen, Carlsbad, CA, USA). Statistical significance was determined by Student’s t test from triplicate experiments (* *p* < 0.05). No significant difference was observed between 1 µg and 2 µg of Ml-LAAOrec DNA.

The pSecTag2B vector containing the Ml-LAAO sequence was synthesized by GenOne Biotechnologies (Rio de janeiro, Brazil) and amplified in *Escherichia. coli* TOP10F. Recombinant Ml-LAAO (Ml-LAAOrec) was then expressed in HEK293 cells under optimized transfection conditions. To monitor efficiency, co-transfection with an EGFP plasmid was performed at a five-fold lower concentration than Ml-LAAOrec DNA, with quantification using a Countess 3FL cell counter (Invitrogen, Carlsbad, CA, USA) (Figure 3A). Different concentrations of Ml-LAAOrec DNA (Figure 3B) and FuGENE^®^ 4K reagent (Promega, Madison, WI, USA) were tested. The optimal condition, consistent with the manufacturer’s instructions, was 1 µg DNA, 3 µL FuGENE (Promega, Madison, WI, USA), and 21 µL Opti-MEM in 24-well plates seeded with 2 × 10^5^ cells at 70–80% confluency, cultured in 500 µL DMEM with 10% FBS.

No significant differences were observed between 1 and 2 µg DNA; therefore, 1 µg was used for subsequent experiments. Transfection efficiency was evaluated by enzymatic activity and confirmed by EGFP expression detected under fluorescence microscopy.

### 2.3. Reverse Transcription Followed by Quantitative Real-Time Polymerase Chain Reaction (qRT-PCR)

**Figure 4:**
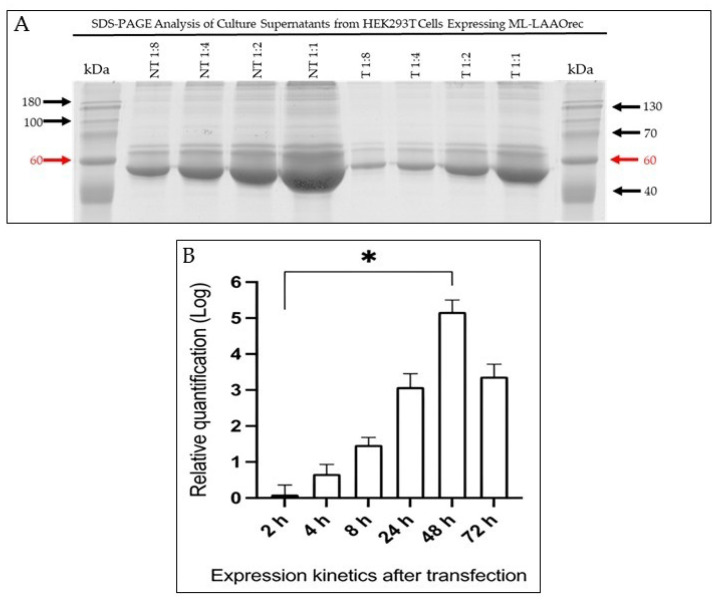
Ml-LAAOrec expression and transcription kinetics in HEK293T cells. (**A**) SDS-PAGE analysis of serial dilutions from culture supernatants collected 24h after transfection. A distinct band at approximately 62 kDa (red arrows), consistent with the expected molecular mass of the Ml-LAAOrec, is visible in transfected (T) and Non-Transfected (NT). However, the intensity of this band suggests co-migration with serum albumin (~66 kDa). Molecular weight marker: Class Five Prestained Multicolor Protein (15–180 kDa; NeoBio). (**B**) Transcription profile of Ml-LAAOrec assessed by qRT-PCR at 2, 4, 8, 24, 48, and 72 h post-transfection. Expression was normalized to RNaseP and quantified via the ΔΔCT method, using 2 h as a baseline. Results are shown as mean ± SD (n = 2). Statistical significance was determined by Dunn’s multiple comparisons test (* *p* < 0.05 vs. 2 h).

No differences were observed in the protein profile between the control (NT) and transfected (T) cells at the expected molecular mass of approximately 62 kDa. This range showed a prominent band at ~60 kDa (red arrow); however, the intensity of this band suggests co-migration with serum albumin (~66 kDa) (Figure 4A). An additional investigation was required to confirm whether mRNA translation was leading to satisfactory expression of the recombinant protein.

To assess the stability of transient expression, Ml-LAAOrec mRNA levels were quantified over a 72 h period post-transfection using qRT-PCR (Figure 4B). The data revealed a progressive increase in mRNA levels up to 48 h, indicating successful transcription. However, a decline in mRNA levels was observed at 72 h, which may reflect the onset of cell death or reduced transcriptional activity.

Quantification was performed using the comparative ΔΔCT method [33] to assess Ml-LAAO mRNA levels at multiple time points, normalized to the endogenous control RNase P. This control aimed to detect potential false negatives caused by RNA degradation and correct for variations in initial sample amounts due to differences in RNA recovery and sample loading, ensuring reliable qRT-PCR data.

### 2.4. Evaluation of Ml-LAAOrec Enzymatic Activity

**Figure 5:**
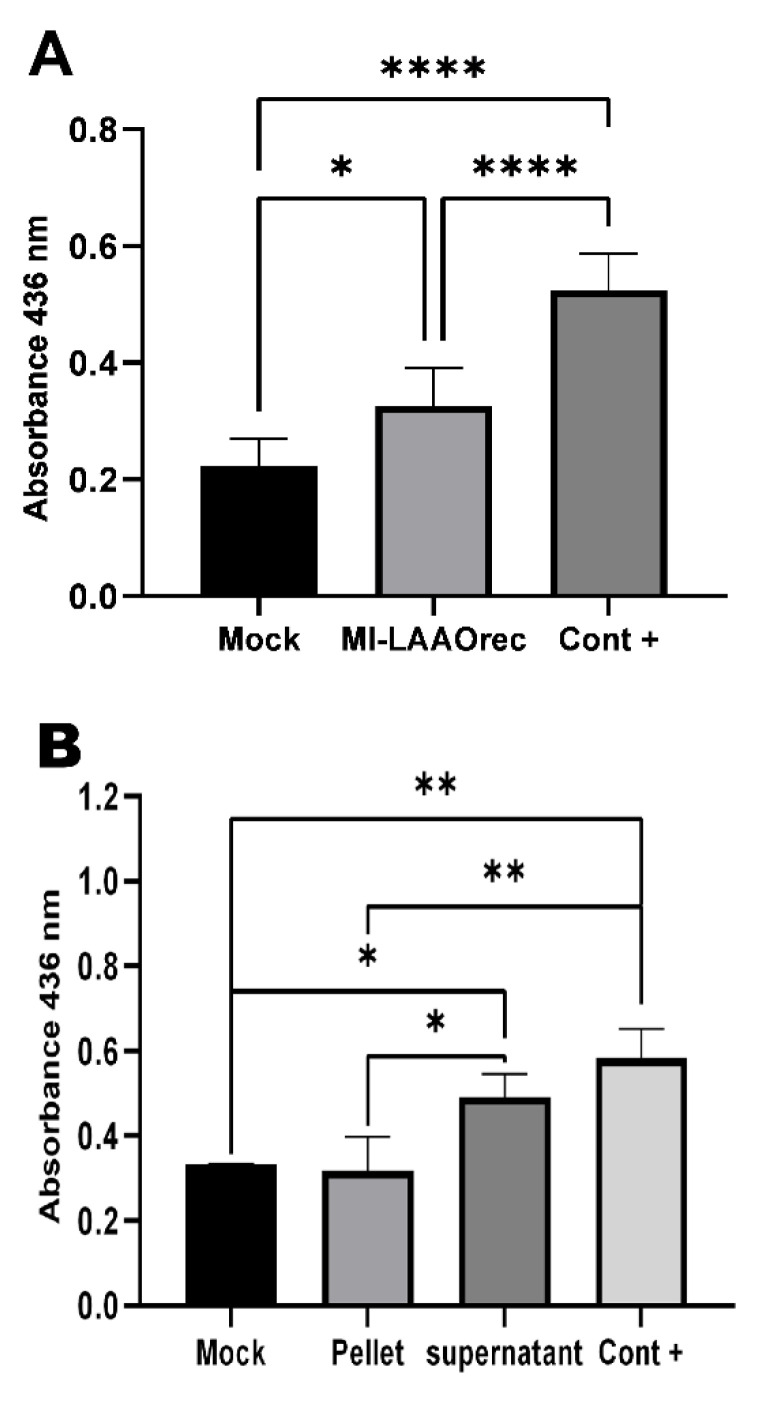
Enzymatic activity of Ml-LAAOrec in transfected HEK293T cells. (**A**) Activity measured in the culture supernatant 24 h after transfection. Mock (black bar): cells transfected without the construct; The Ml-LAAOrec (light gray): recombinant enzyme; Cont+: (dark gray): *Bothrops jararacussu* venom (30 µg/mL; positive control). (**B**) Activity in cellular fractions (Supernatant and pellet). A significant difference was observed for condition (**A**). For condition (**B**), significant differences were detected between Mock and Supernatant, Mock and Cont+, Pellet and Supernatant, Pellet and Cont+. No significant differences were found between Pellet and Mock or between Cont+ and Supernatant. Data are mean ± SD (*n* = 6 for A; *n* = 3 for B). Statistical analysis: one-way ANOVA with Tukey’s post hoc test (* *p* < 0.05, ** *p* < 0.01, **** *p* < 0.0001).

Ml-LAAOrec was successfully expressed in HEK293T cells and showed enzymatic activity. After 24 h of expression, Ml-LAAOrec catalyzed the oxidative deamination activity on L-leucine, confirming its functional expression (Figure 5A). This is consistent with the findings of Kommoju et al. (2007) [34], who successfully expressed an active recombinant LAAO from *Calloselasma rhodostoma* in *Pichia pastoris*.

Ml-LAAOrec was secreted into the culture medium and exhibited catalytic properties like the native venom LAAO (Figure 5B). These results highlight the potential of eukaryotic hosts for proper folding and post-translational modification of LAAOs.

Coutinho et al. 2025 [35] demonstrated that the LAAO activity assay can vary across different venom pools from the same species. Moreover, the enzymatic activity can be influenced by the storage conditions of the venom. For instance, sample solutions maintained at 4 °C for 60 days exhibited a noticeable decline in LAAO activity.

In our present study, we detected LAAO activity only after 24 h of expression. At other time points (48 and 72 h), the activity was not detectable, suggesting a possible time-dependent expression profile or instability of the recombinant enzyme. The underlying causes of this variability need further investigation to determine whether they are related to expression, protein folding, degradation, or assay sensitivity.

### 2.5. Purification and Identification of Ml-LAAOrec

**Figure 6:**
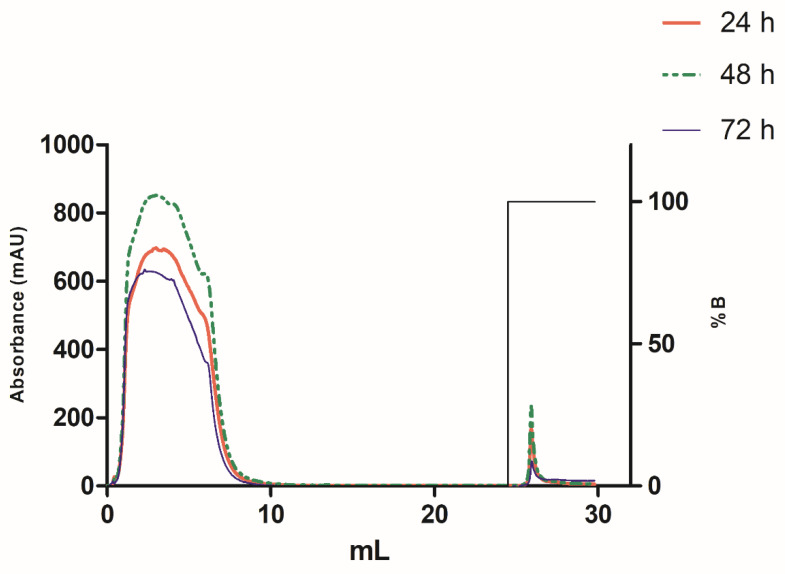
Affinity Chromatography Purification of Ml-LAAOrec. Chromatogram showing the purification of Ml-LAAOrec by affinity chromatography on a nickel column, using supernatants collected at three expression times: 24 h (red line), 48 h (green line), and 72 h (blue line). Non-retained proteins were eluted with the equilibration buffer, while Ml-LAAOrec was eluted with 100% of the elution buffer (B%), as indicated by the black line.

The recombinant enzyme was purified by nickel affinity chromatography from culture supernatants collected at 24, 48, and 72 h post-transfection. In all cases, the Ml-LAAOrec was eluted as a single peak (Figure 6). At 48 h post-transfection, the largest area under the elution curve was detected in the chromatographic profile, indicating a higher concentration of Ml-LAAOrec, consistent with the elevated mRNA levels (Figure 4B).

However, at 72 h, a decrease in Ml-LAAOrec expression was noted. The cause of this reduction remains to be investigated, but this decline in expression is possibly due to medium acidification or H_2_O_2_-induced cellular stress [28,36]. These findings suggest that the 48 h time point is optimal for driving the expression of Ml-LAAOrec, although enzymatic activity in the supernatant was only detected at 24 h post-transfection (Figure 5A).

Samples of Ml-LAAOrec obtained from affinity chromatography were pooled and dialyzed, followed by determination of their protein content by the BCA method, obtaining a total yield of ~350 µg in 20 mL of culture medium. It is important to note that this yield corresponds to a partially purified, LAAO-enriched fraction rather than a highly purified enzyme preparation. In comparison, Kommoju et al. (2007) [34] reported yields of ~0.25–0.5 mg/L of highly purified recombinant LAAO from *Calloselasma rhodostoma* expressed in *Pichia pastoris*, using a two-step purification protocol. Although lower in overall quantity, the present yield reflects an efficient recovery of Ml-LAAOrec from a mammalian expression system in a single purification step, highlighting its potential for the simplified production of recombinant LAAO.

**Figure 7:**
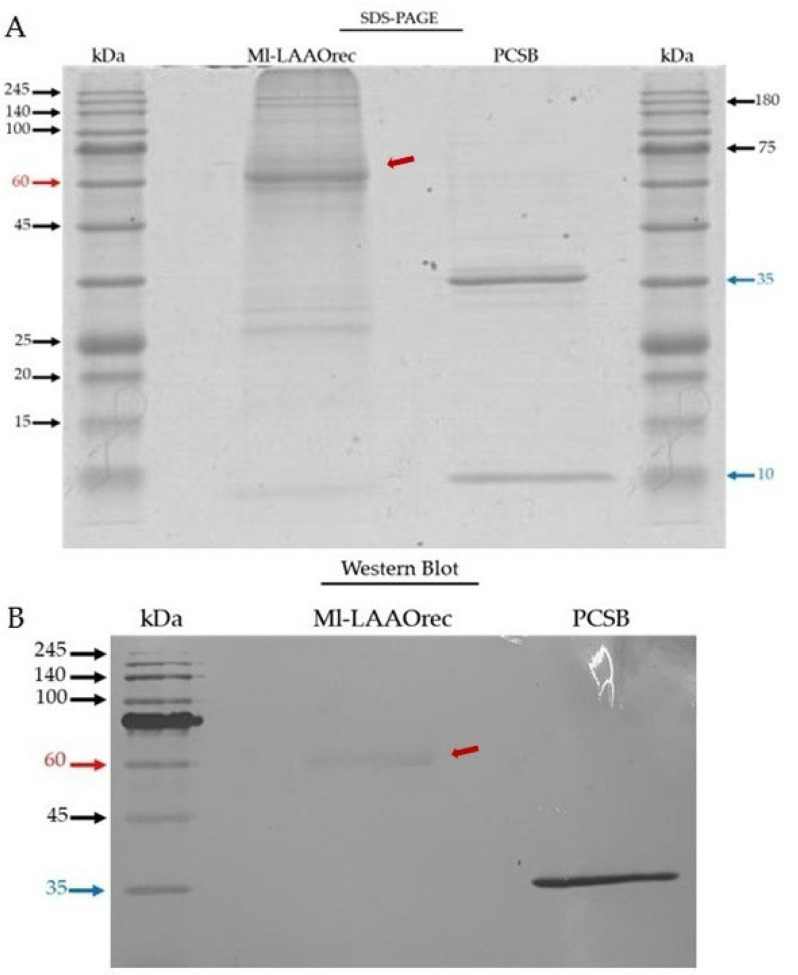
SDS-PAGE and Western blot analysis of recombinant Ml-LAAOrec. (**A**) SDS-PAGE (12%) of lyophilized Ml-LAAOrec after dialysis, showing a prominent band at ~60 kDa (red arrow), close to the predicted mass of Ml-LAAOrec (~62 kDa). The intensity of this band suggests co-migration with serum albumin (~66 kDa). The recombinant protein PCSB (~41.5 kDa, blue arrow) was used as a control. Minor contaminants are observable. (**B**) Western blot using an anti-histidine monoclonal antibody confirmed the presence of His-tagged Ml-LAAOrec and PCSB. The detection of Ml-LAAOrec in the ~60 kDa region supports its successful expression despite co-migration with albumin observed in SDS-PAGE. Molecular mass markers (kDa) are indicated on the left.

The Ml-LAAOrec sample, lyophilized after dialysis, was analyzed by 12% SDS-PAGE electrophoresis (Figure 7A). A prominent band was observed at approximately 60 kDa (red arrow), which is close to the theoretical mass of Ml-LAAOrec (~62 kDa). However, given the high intensity of this band and its proximity to the known molecular weight of serum albumin (~66 kDa), it is likely that albumin co-migrates with Ml-LAAOrec in this region. The presence of serum albumin and additional faint bands are observable, potentially indicative of trace-level contaminants from the culture médium.

To confirm the presence of the recombinant protein, a Western blot was performed using an anti-histidine monoclonal antibody (Figure 7B). The recombinant protein PCSB (~41.5 kDa), previously produced in the laboratory, was included as a positive control. Both the Ml-LAAOrec and PCSB were recognized by the antibody, confirming the expression of His-tagged Ml-LAAOrec in the heterologous system, despite the co-migration with albumin as observed in SDS-PAGE. This suggests that Ml-LAAOrec was successfully expressed in the heterologous system established in this work.

### 2.6. Mass Spectrometry

To further confirm that the expressed protein was Ml-LAAO, an aliquot obtained from affinity chromatography was digested with trypsin, and the resulting peptides were analyzed using LC-MS/MS. The searches were conducted against two databases: *Micrurus lemniscatus* and *Bos taurus*, both constructed using sequences deposited in UniProt. For each database, two separate analyses were performed. In the first analysis, the amino acid sequence of the recombinant protein (Ml-LAAOrec) was included, while in the second, the sequence of the native protein from DDBJ was incorporated.

The results from the *Micrurus lemniscatus* database analysis (Table S1) identified 11 peptides: AGHQVTLLEASESVGGR, FFQPLDLETSADIVINDLSLIHQLPK, IHFEPPLPSNK, RIHFEPPLPSNK, LISEEDLNSAVDHHHHHH, HVVVVGAGIAGLSAAYVLAK, EADYEEFLEIAR, YAMGSITSFTPYQFK, FWEADGIR, SDDIFSYEK, and KVIEELK. Of these, nine peptides were found to be common to both Ml-LAAOrec and Ml-LAAOnat.

Only two peptides, KVIEELK and LISEEDLNSAVDHHHHHH, were found to be exclusive to Ml-LAAOrec (Table S2). The second, LISEEDLNSAVDHHHHHH, corresponds to the C-terminal portion of the polypeptide chain, which includes the histidine tail. This region is specific to Ml-LAAOrec and was introduced during vector construction, supporting the identification of the expressed protein as Ml-LAAO.

The full C-terminal sequence of the recombinant (ARGGPEQKLISEEDLNSAVDHHHHHH) was not identified, as the segment ARGGPEQK was missing. This likely reflects the proteolytic activity of trypsin, which cleaves at the carboxyl side of lysine and arginine residues, generating peptides that may be too short for detection by mass spectrometry. The same idea can be used to explain that the peptide KVIEELK was identified only in Ml-LAAOrec. Saveliev et al. (2013) [37] reported that over 20% of expected cleavage sites remained uncleaved after overnight incubation with trypsin in a yeast protein extract. In this case, the presence of two adjacent tryptic sites may have produced fragments too short to be effectively detected during mass spectrometry analysis. The Ml-LAAOrec identification coverage rate was 27%, corresponding to 144 unique amino acid residues with 100% identity to the original protein sequence from DDBJ, confirming successful expression of the recombinant protein. Concerning the limited 27% sequence coverage, several factors could be attributed to and influence mass spectrometry detection efficiency, such as signal suppression induced by other abundant peptides, undetectable peptides from the same protein, and sample purity. In particular, the high frequency of lysine and arginine residues in the reference sequence attests to the likely generation of low molecular mass peptides.

In addition to Ml-LAAOrec, 104 peptides corresponding to 66 different proteins were identified in the search against the *M. l. lemniscatus* database. Many of these proteins belong to conserved domain families, such as actins, tubulins, histones, ubiquitins, and other proteins associated with structural components and cellular metabolism. Their presence may be attributed to fetal bovine serum (10% FBS) used in the HEK293T cell culture, consistent with contaminants observed in the SDS-PAGE gel electrophoresis (Figure 7A). In fact, to verify the presence of these contaminants, we performed an additional search against the *Bos taurus* database (Table S3), with the MI-LAAOrec sequence incorporated. The results showed the identification of the same conserved proteins observed in the previous analysis, including the exclusive peptides of MI-LAAOrec (KVIEELK and LISEEDLNSAVDHHHHHH).

A homology comparison between *M. l. lemniscatus* and *Bos taurus* databases revealed that 106 of the 115 peptides from *M. l. lemniscatus* (92%) were shared with those of *Bos taurus* (Figure S2). These findings reinforced the idea that these proteins originated from the culture medium. Therefore, additional steps are necessary to improve the purification methods in order to obtain a more homogeneous preparation Ml-LAAOrec. Despite the presence of culture medium contaminants, these analyses confirmed the identification of Ml-LAAOrec peptides in HEK293T cells, further attesting the successful heterologous expression of this enzyme in a eukaryotic system.

## 3. Conclusions

This study reports, for the first time, the successful heterologous expression of the snake venom enzyme L-amino acid oxidase (Ml-LAAO) from *Micrurus lemniscatus* in mammalian cells. The recombinant enzyme (Ml-LAAOrec) was expressed in HEK293T cells, functionally validated by oxidative deamination of L-leucine, and confirmed through molecular and proteomic analyses. These results provide a foundation for future functional studies and biotechnological applications of Ml-LAAO.

## 4. Materials and Methods

### 4.1. Obtaining the Amino Acid Sequence of Ml-LAAO

The amino acid sequence of Ml-LAAO from *Micrurus lemniscatus* was initially inferred by comparing 52-residue peptide fragments, which were previously identified in our lab by Soares (2020): READYEEFLEIAR, NDMEGWYVNLGPMR, LSEFVQENENAWYYIK, and FWEVHGIR, with sequences in the DDBJ (DNA Data Bank of Japan) and UniProt databases using the descriptor ‘*Micrurus lemniscatus lemniscatus*.’ The most similar sequence was identified from the study of Aird et al. (2017) [31], leading to the identification of a full-length sequence consisting of 501 amino acid residues. This sequence (DR043240) and MlLAAO fragments were submitted to the MAFFT software (v7.511) to comparison. The strategy used was to adjust direction according to the first sequence.

### 4.2. In Silico Analysis

Subsequently, an in silico analysis was conducted to predict the physicochemical characteristics of Ml-LAAO, including its molecular weight and theoretical isoelectric point, using the ProtParam software “https://web.expasy.org/protparam/ (accessed on 26 March 2022)”.

The presence of a signal peptide was assessed for two different tools SignalP version 6.0 “https://services.healthtech.dtu.dk/services/SignalP-6.0/ (accessed on 27 April 2024)” and Protter version 1.0 (https://wlab.ethz.ch/protter/start/) (accessed on 11 December 2023)” while conserved protein domains were identified through Pfam version 35.0 “http://pfam.xfam.org/ (accessed on 9 June 2023)”. Secondary structure predictions were carried out using PredictProtein version 2000.02 “https://predictprotein.org/ (accessed on 30 April 2023)” and molecular homology modeling was performed with SWISS-MODEL version 2.3 “http://swissmodel.expasy.org/ (accessed on 29 April 2023)” and Phyre2 version 2.0 “http://www.sbg.bio.ic.ac.uk/phyre2/html/page.cgi?id=index (accessed on 29 April 2023)”. Additionally, potential glycosylation sites were identified using NetNGlyc version 1.0 “https://services.healthtech.dtu.dk/services/NetNGlyc-1.0/ (accessed on 27 March 2023)” and subsequently predicted with Protter.

### 4.3. Ml-LAAO Expression

#### 4.3.1. Plasmid Construction and Amplification

The Ml-LAAO coding sequence was reverse translated from its amino acid sequence using EMBOSS Backtranseq (https://www.ebi.ac.uk/jdispatcher/st/emboss_backtranseq) and codon-optimized for mammalian expression. The sequence was synthesized (GenOne Biotechnologies) and cloned into the pSecTag2B vector (Invitrogen) between HindIII and XhoI sites. Vector features included a CMV promoter, secretion signal from the V-J2-C region of the mouse IgG kappa chain, c-myc epitope, a hexahistidine (6xHis) tag, ampicillin resistance, and a pUC origin of replication. Plasmids were amplified in E. coli TOP10F.

#### 4.3.2. Cell Culture and Transfection

HEK293T cells were maintained in high-glucose DMEM supplemented with 10% FBS and 1% GlutaMAX at 37 °C and 5% CO_2_. For transfection, 2 × 10^5^ cells/well were seeded in 24-well plates. The optimized protocol used 1 µg plasmid DNA, 3 µL FuGENE^®^ 4K (Promega, Madison, WI, USA), and 21 µL Opti-MEM per well. Transfection mixtures were added directly to cells and incubated for ≥24 h.

#### 4.3.3. Optimization and Efficiency Assessment

DNA (1–2 µg) and FuGENE^®^ (Promega, Madison, WI, USA) concentrations were tested according to the manufacturer’s instructions; 1 µg DNA was selected for subsequent experiments. Transfection efficiency was evaluated by co-transfection with an EGFP reporter plasmid (1:5 ratio to Ml-LAAO DNA) and by enzymatic activity. EGFP expression was confirmed by fluorescence microscopy and quantified with a Countess 3FL cell counter (Invitrogen). Mock-transfected cells (no DNA) were used as controls.

### 4.4. RNA Extraction and Quantitative Reverse Transcription PCR (qRT-PCR)

For one-step quantitative reverse transcription PCR (qRT-PCR), HEK293T cells were transfected as described in Section 2.3. Samples were collected at 2, 4, 8, 24, 48, and 72 h post-transfection and stored at −80 °C. Cells were lysed using a buffer consisting of 1% Triton X-100, 50 mM Tris-HCl (pH 8.5), 120 mM NaCl, and 5 mM EDTA. Total RNA was extracted using the PureLink RNA Mini Kit (Invitrogen), following the manufacturer’s instructions. To remove contamination with plasmid DNA, the extracted RNA was treated with DNase I (RQ1 RNase-Free DNase, Promega) for 1 h at 37 °C prior to use in qRT-PCR. Oligonucleotide primers were designed using NCBI Primer-Blast based on the Ml-LAAO insert sequence. Forward (5′-AAAGAGCACTACCGACCTGC-3′) and reverse (5′-GCCATGATCACGCCAATTCC-3′) primers were selected to amplify an 81-base pair (bp) fragment. As an endogenous control, primers targeting a 64 bp fragment of the human RNAseP gene, as described by the CDC, were used: RnaseP-Forward (5′-AGATTTGGACCTGCGAGCG-3′) and RnaseP-Reverse (5′-GAGCGGCTGTCTCCACAAGT-3′). qRT-PCR reactions were performed on a 7500 Fast Real-Time PCR System (Applied Biosystems, Foster City, CA, USA) using the GoTaq^®^ Probe 1-Step RT-qPCR System (Promega, Madison, WI, USA). Each reaction contained 2 μL of RNA, 5 μL of GoTaq^®^ Probe qPCR Master Mix (2×), 0.2 μL of GoScript™ RT Mix for 1-Step RT-qPCR (50×), 0.4 μL of 50 μM Syto™ 9 Green Fluorescent Nucleic Acid Stain (Invitrogen, Carlsbad, CA, USA), 0.5 μL each of 10 μM forward and reverse primers for Ml-LAAO or RNase P (in separate reactions), and 1.4 μL of nuclease-free water.

Thermal cycling conditions included reverse transcription at 50 °C for 30 min, an initial denaturation at 95 °C for 5 min, followed by 45 cycles of denaturation at 95 °C for 15 s and annealing/extension at 60 °C for 1 min. Following amplification, a melting curve analysis was performed. This involved denaturation at 95 °C for 15 s, cooling to 60 °C for 1 min, and gradual heating at 0.3 °C/s up to 95 °C. Fluorescence signals were recorded at 0.3 °C intervals to accurately determine melting profiles. Each 96-well plate included a no-template control (NTC), in which RNA was replaced with nuclease-free water, to verify the absence of contamination. Relative RNA quantification was performed using the comparative ΔΔCT method, with the 2 h post-transfection sample serving as the reference.

### 4.5. Determination of Ml-LAAOrec Activity

The enzymatic activity of Ml-LAAOrec was evaluated in both the culture supernatant and the cell lysate, following the protocols described by Ponnudurai (1994) [38] and Soares (2020) [28], with modifications. The standard reaction mixture (200 µL) included 10 µL of horseradish peroxidase (1 mg/mL) and 60 µL of o-phenylenediamine (OPD, 0.1 mg/mL), preincubated in a 96-well microplate at 37 °C. To this mixture was added 20 µL of the substrate L-leucine (1.0 mM), Tris-HCl buffer (100 mM, pH 8.5), combined with DMEM culture medium (control), or *Bothrops jararacussu* venom (30 µg/mL) as a control, or Ml-LAAOrec obtained from the DMEM culture medium after expression. The enzymatic reaction was carried out at 37 °C for 30 min, and stopped by adding 20 µL of acetic acid (60% *v*/*v*). The resulting chromophore was measured at a wavelength of 436 nm using a Multiskan Sky microplate reader (Thermo Scientific, Singapore).

### 4.6. Ml-LAAOrec Purification and Quantification

Ml-LAAOrec was purified through affinity chromatography (HisTrap HP column (7 × 25 mm, ~1 mL resin volume; 6% highly cross-linked agarose resin, GE Healthcare Uppsala, Uppsala, Sweden), using an HPLC system (ÄKTA purifier 900, GE Healthcare). Aliquots of 2 mL of DMEM culture medium were filtered (Millex-HA (Darmstadt, Hesse, Germany) 0.45 µm membrane filter) and loaded onto the equilibrated column. The elution was performed with an imidazole buffer 0.005–1 M imidazole in 0.02 M sodium phosphate, 0.5 M NaCl, pH 7.4). Eluted fractions were monitored at 280 nm to detect protein peaks.

The Ml-LAAOrec fractions were subsequently concentrated and desalted through dialysis using a Tubing Cellulose membrane with an 8000 MWCO and the sample was finally maintained in Milli-Q water, lyophilized, and stored at −20 °C.

The protein concentration of the dialyzed samples was determined using the Bicinchoninic Acid (BCA) assay as described by Smith et al. (1985). The QuantPro™ BCA Assay Kit (LOT #SLCG1309, Sigma-Aldrich Darmstadt, Hesse, Germany) was employed, with bovine serum albumin (BSA) as the standard. Measurements were performed in triplicate, and the standard curve showed a correlation coefficient of R^2^ = 0.9873.

### 4.7. SDS-PAGE and Western Blot Assay

The protein profile of Ml-LAAOrec was analyzed using polyacrylamide gel electrophoresis (PAGE) based on the Laemmli method (1970) [39]. A separation gel containing 12% polyacrylamide was overlaid with a stacking gel containing 4% polyacrylamide. Electrophoresis was conducted at 30 mA for approximately 2 h at room temperature. Approximately 50 µg of protein were loaded into each gel lane for both pre- and post-purification samples. The samples were prepared without a reducing agent. After electrophoresis, the gel was stained with Coomassie Brilliant Blue R-250 and destained. A 10–245 kDa protein ladder (True Color High Range Protein Marker, Sinapse Inc., Belo Horizonte, Minas Gerais, Brazil.) or 15–180 kDa Class Five Prestained Multicolor Protein (NeoBio, Botucatu, São Paulo, Brazil.) were used as a molecular weight standard. As a positive control, the recombinant protein PCSB (~41.5 kDa), kindly provided by Dr. Janete Soares (Laboratório de Biologia Celular, Funed, Belo Horizonte, Minas Gerais, Brazil), was included in the gel.

For Western blot analysis, the gel was transferred onto a nitrocellulose membrane (Bio-Rad, Munich, Bavaria, Germany). The membrane was blocked for 1 h at room temperature with 1% bovine serum albumin (BSA) prepared in a PBS buffer containing 0.1% Tween-20 (PBS-T). Subsequently, the membrane was incubated with an anti-His antibody (BD Biosciences, San Jose, CA, USA) at a 1:2000 dilution in PBS-T for 2 h. Following four washes with PBS-T, the membrane was incubated with peroxidase-conjugated protein G (Sigma-Aldrich, Darmstadt, Hesse, Germany.) diluted 1:2500 in PBS-T for 1 h. Protein bands were visualized using chromogenic substrate. The PCSB protein was also included in the blot as a positive control for anti-His-tag antibody recognition.

### 4.8. Sample Preparation (In-Solution Digestion) for Nano Liquid Chromatography (Nano-LC) Coupled to Electrospray Ionization Spectrometry (ESI)

After dialysis, 50 µg of Ml-LAAOrec was solubilized in Milli-Q water and precipitated by incubation with 25% TCA at 4 °C for 12 h. The resulting precipitate was washed with 500 µL of cold acetone and dried. Following solvent evaporation, the pellet was dissolved in 60 µL of 8 M urea. Reduction was performed with 10 mM Tris(2-carboxyethyl)phosphine (TCEP, Rockford, IL, USA), followed by alkylation with 100 mM iodoacetamide (Freiburg, Baden-Württemberg, Germany). Thereafter, 125 µL of ultrapure water, 15 µL of 1M ammonium bicarbonate containing 10% (*v*/*v*) of 100 mM CaCl_2_ and Trypsin (Promega Corporation, Madison, WI, USA) at a 1:50 (*w*/*w*) enzyme-to-protein ratio were added, and digestion was carried out overnight at 37 °C. The reaction was stopped with 1% trifluoroacetic acid (TFA), and the peptides were desalted using Sep-Pak C18 cartridges (Milford, MA, USA). The eluted peptides were lyophilized and stored at −80 °C until analysis.

The trypsinized sample was analyzed by nano-LC-MS/MS using a nanoUHPLC Ultimate^®^ 3000 system (Dionex, Thermo Fisher Scientific, Germany) coupled to a Q Exactive mass spectrometer (Thermo Scientific). Peptides were separated on a Nano-Trap Acclaim PepMap100 C18 column and subsequently on a C18 capillary column. The mobile phase consisted of 0.1% formic acid (solution A) and 80% acetonitrile (solution B). Elution was performed at a constant flow rate of 0.3 µL/min using a gradient of solution B (0–99%). Peptides were detected in positive mode at 3.45 kV and 250 °C. Spectra were acquired at an MS1 resolution of 70,000 and an MS/MS resolution of 35,000, using HCD for fragmentation.

### 4.9. Search for Identities

Precursor ions (MS) were processed using PEAKS Studio software (Version 8.5) and analyzed against two protein sequence databases downloaded from UniProt in February 2024. The first database, containing 25,137 sequences, was constructed using “*Micrurus lemniscatus lemniscatus*” as the descriptor, while the second database, comprising 47,131 sequences, was generated using “*Bos taurus*” as the descriptor. The latter database was included to assess potential contaminants from fetal bovine serum used in cell culture.

Additionally, database searches were conducted twice. In the first case, the Ml-LAAOrec sequence was manually incorporated into each database. In the second, Ml-LAAOrec sequence was substituted with the native sequence, as originally deposited in the DNA Data Bank of Japan (DDBJ).

The search parameters applied to all three databases were as follows: allowance for up to two missed cleavage sites by trypsin, a fragment mass error tolerance of 0.1 Da, and a precursor mass error tolerance of 10.0 ppm. Carbamidomethylation of cysteine residues (+57.02 Da) was specified as a fixed modification, while methionine oxidation (+15.99 Da) was included as a variable modification. Spectral data were analyzed against both databases, and peptide homology results were processed using PEAKS Studio version 8.5.

## Figures and Tables

**Table 1:** Predicted physicochemical properties of Native and Recombinant Ml-LAAO (ProtParam Analysis).

Properties	Ml-LAAOnat	Ml-LAAOrec
Number of nucleotides	1503	1629
Number of amino acids	501	543
Molecular weight	~57 kDa	~62 kDa
Theoretical Isoelectric Point	9.02	8.97
Half-life estimate (in vitro, in mammalian cells)	30 h	30 h
Instability index	33.17 (stable)	35.55 (stable)
Aliphatic index	83.71	83.54

## Data Availability

The original contributions presented in this study are included in the article and Supplementary Materials. Further inquiries can be directed to the corresponding author.

## Supplementary Materials

The following supporting information can be downloaded at: https://www.mdpi.com/article/10.3390/toxins17100491/s1, Figure S1: In silico prediction of the signal peptide and topology of the native Ml-LAAO protein (Ml-LAAOnat); Figure S2: Venn Diagram of *Bos taurus* and *M. l. lemniscatus;* Table S1: Peptide-Protein List M. l. lemniscatus—Ml-LAAOnat; Table S2: Peptide-Protein List M. l. lemniscatus—Ml-LAAOrec; Table S3: Peptide-Protein List Bos Taurus.

## Abbreviations

LAAO	L-amino acid oxidase
Ml-LAAO	L-amino acid oxidase from *Micrurus lemniscatus*
Ml-LAAOnat	Native Ml-LAAO
Ml-LAAOrec	Recombinant Ml-LAAO
DMEM	Dulbecco’s Modified Eagle’s Medium
EGFP	Green Fluorescent Protein
PBS	Phosphate-buffered saline
SDS-PAGE	Sodium dodecyl sulfate–polyacrylamide gel electrophoresis
HPLC	High-performance liquid chromatography
DDBJ	DNA Data Bank of Japan
qRT-PCR	Quantitative reverse transcription polymerase chain reaction
RNAse P	Ribonuclease P (endogenous control)
FBS	Fetal bovine serum
OPD	o-Phenylenediamine
BSA	Bovine serum albumin
TFA	Trifluoroacetic acid
TCEP	Tris(2-carboxyethyl)phosphine
NTC	No template control
ESI	Electrospray ionization
LC-MS/MS	Liquid chromatography tandem mass spectrometry
MS/MS	Tandem mass spectrometry
PBS-T	PBS with Tween-20
H_2_O_2_	Hydrogen peroxide
HCD	Higher-energy collisional dissociation
