# Supplementary material for: Expression of L-Amino Acid Oxidase (Ml-LAAO) from the Venom of the Micrurus lemniscatus Snake in a Mammalian Cell System
Source: Toxins (Basel). 2025 Oct 2;17(10):491. doi: 10.3390/toxins17100491 (PMC12567790; doi:10.3390/toxins17100491)
Supplement: Supplementary file 1 [file toxins-17-00491-s001.zip › S1 - Phyre2 final.pdf]

Return to main results | Retrieve Phyre Job Id [ ] Fetch

Phyre2.2

Job Description	Undefined		
Confidence	100.00%	Date	Sat Jul 5 19:20:11 BST 2025
Rank	4	Aligned Residues	480
% Identity	76%	Template	c2iidD_
PDB info	**PDB header:** oxidoreductase	**Chain:** D: **PDB Molecule:** L-amino-acid oxidase	**PDBTitle:** structure of l-amino acid oxidase from calloselasma rhodostoma in complex with l-phenylalanine **PDB Entry:** PDBe RCSB PDBj
Resolution	UNK		
Model Dimensions (Å)	**X**:67.414 **Y**:58.516 **Z**:64.129		

Show / Hide SS confidence

Show / Hide Conservation and Alignment quality

Insertion relative to template

Deletion relative to template

Catalytic residue from the CSA

Detailed help on interpreting your alignment

	22 . . . . 30 . . . . 40 . . . . 50 . . . . 60 . . . . 70 . . . .
Predicted Secondary structure	
Query Sequence	R RPL GECFR E ADYEEF LEI A R NG LKKTSN P KHV VVVGAG I AGLSAAYVL A KAGHQV
Template Sequence	R NPL AECFQ E NDYEEF LEI A R NG LKATSN P KHV VI VGAG M AGLSAAYVL A GAGHQV
Template Known Secondary structure	TT GGG G T T S SS B TT
Template Predicted Secondary structure	

	82 . . . . 90 . . . . 100. . . . . 110. . . . . 120. . . . . 130. . . . . . .
Predicted Secondary structure	
Query Sequence	A SES VGGRV K TYRNDMEGW Y V NL GPMRLP E RHR I VREYI R KF RLPLSEF V QENENA
Template Sequence	A SERPGGRV R TYRNEE AGW Y A NL GPMRL P E KHR I VREYI R KF DLRLNEF S QENDNA
Template Known Secondary structure	S SSS SBT T TT TT S S T T T T S TTS
Template Predicted Secondary structure	

	142. . . . . . . 150. . . . . 160. . . . . 170. . . . . 180. . . . . 190. . . . . . .
Predicted Secondary structure	
Query Sequence	K NI RKKVSE V KNNPGHFKY P V KP SEKGKS A SQL YQESLR K VI EELKMTN C SYI LNK
Template Sequence	K NI RKKVGE V KKDPGL LKY P V KP SEAGKS A GQL YEESLG K VV EELKRTN C SYI LNK
Template Known Secondary structure	T T GGG G G GGT T T S
Template Predicted Secondary structure	

	202. . . . . . . 210. . . . . 220. . . . . 230. . . . . 240. . . . . 250. . . . . . .
Predicted Secondary structure	
Query Sequence	S TKE YLI KE G NLSPGA VDM I G DL LNE ESS F YLS FI ESLK S DDI FSYE KR F DEI VGC
Template Sequence	S TKE YLI KE G DLSPGA VDM I G DL LNE DSG Y YVS FI ESLK H DDI FAYE KR F DEI VDC
Template Known Secondary structure	B T S TT GGG T TSB TT TT
Template Predicted Secondary structure	

	262. . . . . . . 270. . . . . 280. . . . . 290. . . . . 300. . . . . 310. . . . . .
Predicted Secondary structure	
Query Sequence	P KSMYQAI A E MVHLNAQVI K I QN NRGNVR V TYQ TP . AKT S SVTADYVI V C STSRAA
Template Sequence	P TAMYRDI Q D KVHFNAQVI K I QQ NDQKVT V VYE TLSKET P SVTADYVI V C TTSRAV
Template Known Secondary structure	TG G G SS SS SSS S S
Template Predicted Secondary structure	

	321. . . . . 330. . . . . 340. . . . . 350. . . . . 360. . . . . 370. . . . . .
Predicted Secondary structure	
Query Sequence	F EPPLPS NK A RALRSI HYR S A I K I FL TCT N RFWEADGI R G GKSTTDLPS R FI YYFN
Template Sequence	F NPPLLP KK A HALRSVHYR S G T K I FL TCT T KFWEDDGI H G GKSTTDLPS R FI YYPN
Template Known Secondary structure	S S S GG GGGT S S SS TT S
Template Predicted Secondary structure	

	381. . . . . 390. . . . . 400. . . . . 410. . . . . 420. . . . . 430. . . . . .
Predicted Secondary structure	
Query Sequence	N GI GVI MAY V L ADDAKFFQ P L DL ETS ADI V I NDLSLI HQ L PKKKI QDLC R ASLI QK
Template Sequence	N GVGVI I AY G I GDDANFFQ A L DF KDC ADI V FNDLSLI HQ L PKKDI QS FC Y PSVI QK
Template Known Secondary structure	T S TTTT T S T
Template Predicted Secondary structure	

	441. . . . . 450. . . . . 460. . . . . 470. . . . . 480. . . . . 490. . . . . .
Predicted Secondary structure	
Query Sequence	K YAMGSI TS F TPYQFKNYF E T VA EPVGRI Y FAGEYTAKD H GWI DSTI KS G LRAARD
Template Sequence	K YAMGGI TT F TPYQFQHFS D P LT ASQGRI Y FAGEYTAQA H GWI DSTI KS G LRAARD
Template Known Secondary structure	T TT SS TT BTT S GGGSSS S S
Template Predicted Secondary structure	

	501
Predicted Secondary structure	
Query Sequence	S
Template Sequence	S
Template Known Secondary structure	
Template Predicted Secondary structure	

Download: Text version FASTA pairwise alignment 3D Model in PDB format

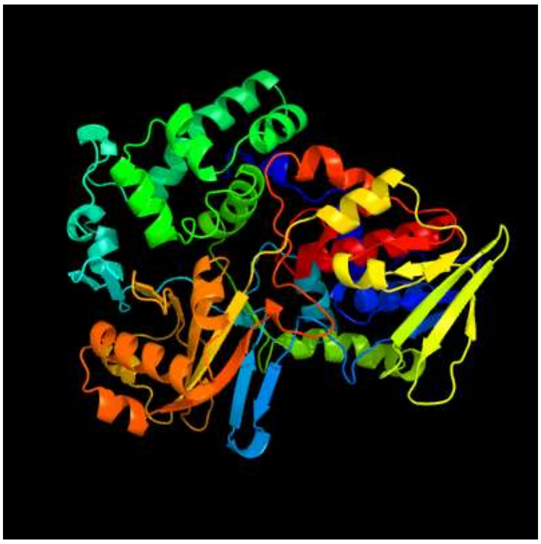

View in JSmol

Send structure to FirstGlance for more viewing options

Phyre is now FREE for commercial users!

All images and data generated by Phyre2 are free to use in any publication with acknowledgement

Accessibility Statement

Please cite: Phyre2.2: A Community Resource for Template-based Protein Structure Prediction
Powell HR *et al.* Journal of Molecular Biology (2025) *in press* DOI: https://doi.org/10.1016/j.jmb.2025.168960

© Structural Bioinformatics Group, Imperial College, London
Michael Sternberg
Disclaimer
Terms and Conditions

Phyre2 is part of **Genome3D**
