# Supplementary material for: Expression of L-Amino Acid Oxidase (Ml-LAAO) from the Venom of the Micrurus lemniscatus Snake in a Mammalian Cell System
Source: Toxins (Basel). 2025 Oct 2;17(10):491. doi: 10.3390/toxins17100491 (PMC12567790; doi:10.3390/toxins17100491)
Supplement: Supplementary file 1 [file toxins-17-00491-s001.zip › toxins-3843476-Supplementary.pdf]

toxins

MDPI

Article

Expression of L-Amino Acid Oxidase (Ml-LAAO) from the Venom of the *Micrurus lemniscatus* Snake in a Mammalian Cell System

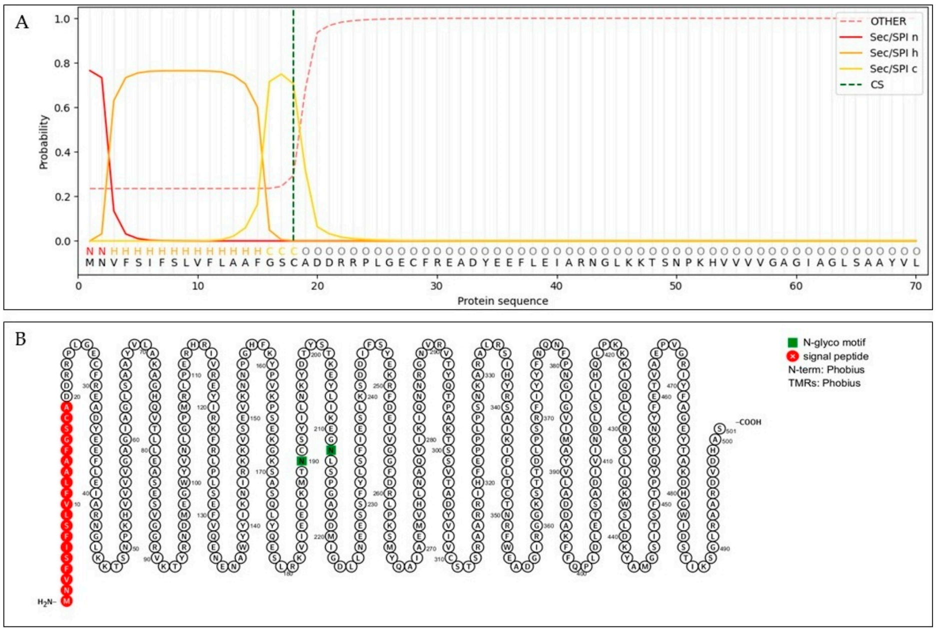

Figure S2. ***In silico*** **prediction of the signal peptide and topology of the native Ml-LAAO protein (Ml-LAAOnat).** (A) Signal peptide prediction using the SignalP 6.0 server. The algorithm identified a signal peptide with high confidence and predicted a cleavage site between residues Cys18 and Ala19 (cleavage site probability: 0.987), consistent with secretion via the Sec/SPI pathway. This pathway involves the recognition of the N-terminal signal peptide by the Sec translocon machinery, followed by cleavage by signal peptidase I (SPI), enabling the transport of the protein across the membrane and its release in a mature, functional form. (B) Topological analysis using the Protter tool. The signal peptide (in red) is located at the N-terminus, and potential N-glycosylation motifs (in green) are distributed along the sequence, confirming a topology consistent with a secreted, non-membrane-bound protein.

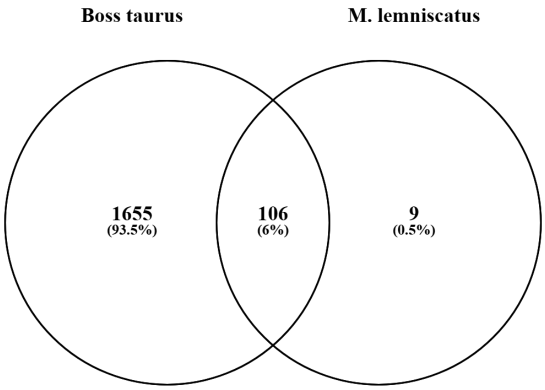

Figure S3. Venn Diagram of *Bos taurus* and *M. l. lemniscatus.* Comparison of peptides identified by mass spectrometry and registered in the *Bos taurus* and *M. lemniscatus* databases. The intersection of the sets represents the 106 common peptides identified in both databases.
